# RING Finger Protein 38 Is a Neuronal Protein in the Brain of Nile Tilapia, *Oreochromis niloticus*

**DOI:** 10.3389/fnana.2017.00072

**Published:** 2017-08-31

**Authors:** Kai Lin Cham, Tomoko Soga, Ishwar S. Parhar

**Affiliations:** Brain Research Institute, School of Medicine and Health Sciences, Monash University Bandar Sunway, Malaysia

**Keywords:** immunocytochemistry, *in situ* hybridisation, midbrain, preoptic area, brain

## Abstract

Really interesting new gene (RING) finger protein is a type of zinc-binding motif found in a large family of functionally distinct proteins. RING finger proteins are involved in diverse cellular processes including apoptosis, DNA repair, cell cycle, signal transduction, tumour suppressor, vesicular transport, and peroxisomal biogenesis. RING finger protein 38 (RNF38) is a member of the family whose functions remain unknown. To gain insight into the putative effects of RNF38 in the central nervous system, we localised its expression. The aim of this study was to identify the neuroanatomical location(s) of *rnf38* mRNA and its peptide, determine the type of RNF38-expressing cells, and measure *rnf38* gene expression in the brain of male tilapia. The distributions of *rnf38* mRNA and its peptide were visualised using *in situ* hybridisation with digoxigenin-labelled RNA antisense and immunocytochemistry, respectively. Both were identically distributed throughout the brain, including the telencephalon, preoptic area, optic tectum, hypothalamus, cerebellum, and the hindbrain. Double-labelling immunocytochemistry for RNF38 and the neuronal marker HuC/D showed that most but not all RNF38 protein was expressed in neuronal nuclei. Quantitative real-time polymerase chain reaction showed the highest level of *rnf38* mRNA in the midbrain, followed by the preoptic area, cerebellum, optic tectum, telencephalon, hindbrain and hypothalamus. These findings reveal a differential spatial pattern of RNF38 in the tilapia brain, suggesting that it has potentially diverse functions related to neuronal activity.

## Introduction

Really interesting new gene (RING) finger domain is a zinc-binding motif found in a large family of functionally distinct proteins (Eisenberg et al., 2002). RING finger proteins are involved in diverse cellular functions such as regulating apoptosis (cIAP1, MDM2, and SIAH1) (Roperch et al., 1999; Hu and Yang, 2003; Kojima et al., 2005), cell cycle (APC11 and RBX1) (Gmachl et al., 2000; Jia et al., 2009), DNA repair (BRCA1, RAD5, and RAD18) (Gowen et al., 1998; Ulrich and Jentsch, 2000), signal transduction (TRAF2) (Vallabhapurapu et al., 2008), tumour suppression (BRCA1, CBL, and SIAH1) (Roperch et al., 1999; Shakya et al., 2008; Sanada et al., 2009), vesicular transport (PEP5 and RMA1) (Srivastava et al., 2000; Matsuda et al., 2001), and peroxisomal biogenesis (PEX2 and PEX12) (Platta et al., 2009). Proteins containing the RING finger motif are capable of mediating ubiquitination and thus, act as E3 ubiquitin ligases. Ubiquitination is achieved when ubiquitin is covalently attached to proteins via an enzymatic cascade involving ubiquitin-activating enzyme (E1), ubiquitin-conjugating enzyme (E2), and ubiquitin-ligating enzyme (E3) (Wilkinson, 1987; Deshaies and Joazeiro, 2009). However, not all RING-containing proteins are E3 ubiquitin ligases.

RING finger protein 38 (RNF38) possesses a conserved RING-H2 domain among species, which consists of Cys-X_2_-Cys-X_(9−39)_-Cys-X_(1−3)_-His-X_(2−3)_-His-X_2_-Cys-X_(4−48)_-Cys-X_2_-Cys, where Cys is a cysteine residue, His is a histidine residue and X is any amino acid residue and binds two zinc atoms (Eisenberg et al., 2002). *Rnf38* mRNA is widely expressed in the brain, heart, liver, spleen, placenta, and testis (Eisenberg et al., 2002), and its protein is a nuclear protein that can bind to and ubiquitinate tumour-suppressor protein p53 (Sheren and Kassenbrock, 2013) through its E3 ubiquitin ligase activity (Sheren and Kassenbrock, 2013; Buetow et al., 2015). RNF38 is often associated with ageing and cancerous diseases (Fu et al., 2006; Nacheva et al., 2010; Sato et al., 2013). For examples, *rnf38* is up-regulated in the hypothalamic area during ageing (Fu et al., 2006) and concomitantly deleted during the lymphoid blast transformation of chronic myeloid leukaemia (Nacheva et al., 2010).

RING finger proteins such as antidepressant-related gene 34 (ADRG34) and RING finger protein 180 (RNF180/RINES) are modulated by the serotonergic system (Yamada et al., 2000; Kabayama et al., 2013), which in turn regulates emotional and social behaviour (Kabayama et al., 2013). Recently, chronic treatment of the medaka fish with the antidepressant citalopram up-regulated brain expression of *rnf38* (Moriya et al., 2015), while similar treatment of male mice yielded comparable results (Soga et al., unpublished observations). Thus, there is evidence of a relationship between RNF38 and the vertebrate serotonin system, which could influence social behaviour.

The Nile tilapia, *Oreochromis niloticus*, is an excellent model organism to investigate the role of RING finger proteins in social behaviour as each individual male fish exhibits a distinctive phenotype in the form of dominance and subordination. To gain insight into the putative regional influence of RNF38 in the central nervous system, we localised its expression in the brain. *Rnf38* mRNA and protein were identified using *in situ* hybridisation (ISH) and immunocytochemistry (ICC), respectively, and double-labelling immunocytochemistry (d-ICC) was performed to determine the type of RNF38-expressing cells. Absolute *rnf38* mRNA levels were examined using real-time polymerase chain reaction (PCR) to quantitatively verify the observed regional expression of ISH.

## Materials and methods

### Animals

Sexually mature male Nile tilapia were maintained in freshwater aquaria at 28 ± 0.5°C under a controlled natural light regime (14-h light; 10-h dark cycle) in standard fish tanks (size: 450 × 295 × 300 mm) equipped with circulating water systems and constant aeration. The fish were fed twice a day with cichlid pellets (Ziegler, Gardners, PA, USA). All experimental procedures were conducted in compliance with the guidelines of Monash University Animal Ethics Committee, AEC (MARP/2015/109).

### Localisation of *rnf38* mRNA

#### RNA probe synthesis

The primers for RNA probe synthesis were designed to amplify a 300 bp fragment of tilapia *rnf38* (GenBank accession number XM 005467588.3) from whole brain cDNA. The primer sequences were as follows: forward primer, 5′-CTTGGCGAGCATCTGTCTT-3′, and reverse primer, 5′-TTCCACTTCACCATCATCGA-3′. The resulting fragment was cloned into a pGEM-T Easy Vector (Promega, Madison, WI, USA) to obtain pGEM T-Easy/*rnf38* plasmid and the sequence of the cDNA-containing plasmid was confirmed by sequencing using BigDye Terminator v3.1 Cycle Sequencing kit (Applied Biosystems, Foster City, CA, USA) and 3310 Genetic Analyzer (Applied Biosystems). After confirming the sequence of the cloned tilapia *rnf38* fragment (300 bp; position 2490-2789), the cDNA-containing plasmid was linearised with either *Sal1* or *Bsp191* restriction endonuclease and purified using Wizard SV Gel and PCR Clean-up System (Promega). Anti-sense and sense tilapia *rnf38* riboprobes were synthesised from the purified linearised plasmid using MAXIscript *In Vitro* Transcription Kit (Ambion, Austin, TX, USA) and labelled using digoxigenin (DIG)-RNA labelling mix (Roche Diagnostics, Basel, Switzerland). The transcription mixture (10 μL) consisted of 0.5 μg linearised plasmid, ATP, CTP, and GTP (1 mM each), 0.65 mM UTP, 0.35 mM DIG-UTP, 10 mM DTT, 1 U/μL RNase Inhibitor, and T7 or SP6 RNA polymerase (0.9 or 0.95 U/μL respectively) was incubated at 37°C for 2 h. The digestive reactions were halted by the addition of 20 mM EDTA (pH 8.0), and synthesised riboprobes were purified through two precipitation steps by adding 400 mM and 37.5 μL of 100% ethanol. The riboprobes were stored at −80°C until use.

#### Brain tissue preparation for ISH

Sexually mature males (*n* = 3) were anaesthetised by immersion into 0.02% benzocaine solution (Sigma, St. Louis, MO, USA) prior to decapitation. The brains then were harvested and fixed in 4% paraformaldehyde in 0.1 M phosphate buffer (pH 7.3) for 6 h at 4°C, cryoprotected in 20% sucrose in 0.1 M phosphate buffer (pH 7.3), and embedded in frozen section compound (Leica, Wetzlar, Germany). Brain sections were sectioned in coronal plane (15 μm) using a cryostat (Leica CM1860), thaw-mounted onto silane-coated slide glass (Muto Pure Chemicals, Tokyo, Japan) and stored at −80°C until use for DIG ISH.

#### ISH

Brain sections were permeabilised with 0.2M HCl for 10 min, treated with proteinase K (1 μg/mL) for 15 min at 37°C, and hybridised with DIG-labelled anti-sense RNA probe (50 ng/mL) for *rnf38* overnight at 55°C in a closed moist chamber. An equivalent amount of DIG-labelled sense probe was applied onto alternate sections. The sections were washed stringently after hybridisation with 2X saline sodium citrate (SSC) for 30 min at room temperature, followed by 2X SSC for 30 min at 60°C and 0.1X SSC for 30 min at 60°C, and blocked with 2% normal sheep serum. Alkaline phosphatase-conjugated anti-DIG antibody (1:500, 11093274910, RRID:AB_514497, Roche Diagnostics) was then used to detect the hybridised DIG-labelled RNA probes and 4-nitroblue tetrazolium chloride/5-bromo-4-chloro-3-indolyl-phosphate (NBT/BCIP, Roche Diagnostics) was used to develop the chromogenic reaction.

#### Image analysis

Section images were scanned and captured with a MIRAX MIDI slide scanner (Carl Zeiss, Oberkochen, Germany) and computer software (Pannoramic Scanner; 3DHISTECH, Budapest, Hungary). The staining density of *rnf38* mRNA was subjectively scored on a four-point scale as follows: + + + (high), + + (moderate), + (low), and - (absent). Nomenclature for the brain area was adopted from Parhar (1990); Soga et al. (2005), and Ogawa et al. (2016).

### Localisation of RNF38 protein in the brain

#### Brain tissue preparation for ICC

Sexually mature males (*n* = 2) were anaesthetised by immersion into 0.02% benzocaine solution (Sigma) prior to decapitation. The brains were then harvested and fixed in 4% paraformaldehyde in 0.1 M phosphate buffer (pH 7.3) for 6 h at 4°C, cryoprotected in 20% sucrose in 0.1 M phosphate buffer (pH 7.3), and embedded in frozen section compound (Leica). The brains were sectioned in coronal plane (15 μm) using a cryostat (Leica CM1860) and mounted on silane-coated glass slides (Muto Pure Chemicals) and stored at −80°C until use for ICC.

#### ICC

Primary polyclonal rabbit anti-RNF38 antiserum (1:100, ab121487, RRID:AB_11128227, Abcam, Cambridge, UK) including 0.5% Triton-X and 2% normal goat serum was applied to each section, and the slides were incubated in a closed moist chamber for 48 h at 4°C. Pre-absorption of the primary antiserum with 5 μg/ml RNF38 antigen (VVFSGQHLPVCSVPPPMLQACSVQHLPVPY AAFPPLISSDPFLIHPPHLSPHHPPHLPPPGQFVPFQTQQSRSPLQRIENEVELLGEHLPVGGFTYPPSAHPPTLPPSAPL, ab165637, Abcam) was applied onto alternate sections. The sections were then incubated in biotinylated anti-rabbit immunoglobulin IgG (1:200) for 30 min and avidin-biotinylated horseradish peroxidase complex (1:50) for 45 min at room temperature (PK-6101, RRID:AB_2336820, Vectastain ABC Elite Kit, Vector Laboratories, Burlingame, CA, USA). Antigen-antibody complexes were visualised with Alexa Fluor 488 Streptavidin (1:500; S32354, RRID:AB_2315383, Invitrogen Corporation, Carlsbad, CA, USA).

#### Image analysis

Section images were scanned and captured with a MIRAX MIDI slide scanner (Carl Zeiss) with an appropriate excitation filter for Alexa Fluor 488 and computer software (Pannoramic Scanner). RNF38 peptide staining density was subjectively scored on a four-point scale as follows: + + + (high), + + (moderate), + (low), and – (absent). Nomenclature for the brain area was adopted from Parhar (1990); Soga et al. (2005), and Ogawa et al. (2016).

### Type of RNF38-expressing cells

#### Brain tissue preparation for d-ICC

A sexually mature male (*n* = 1) was anaesthetised by immersion into 0.02% benzocaine solution (Sigma, St. Louis, MO, USA) prior to decapitation. The brain was then harvested and fixed in 4% paraformaldehyde in 0.1 M phosphate buffer (pH 7.3) for 6 h at 4°C, cryoprotected in 20% sucrose in 0.1 M phosphate buffer (pH 7.3), and embedded in frozen section compound (Leica). The brain was sectioned in sagittal plane (15 μm) using a cryostat (Leica CM1860) and mounted on silane-coated glass slides (Muto Pure Chemicals) and stored at −80°C until use for d-ICC.

#### d-ICC of RNF38 with neuronal marker HuC/D or glial fibrillary acidic protein (GFAP)

Primary polyclonal rabbit anti-RNF38 antiserum (1:100, ab121487, RRID:AB_11128227, Abcam) including 0.5% Triton-X and 2% normal goat serum was applied to each section, and the slides were incubated in a closed moist chamber for 48 h at 4°C. The sections then were visualised with goat anti-rabbit IgG secondary antibody, Alexa Fluor 594 conjugated (1.400, A11037, RRID:AB_2534095, Thermo Fisher Scientific, Waltham, MA, USA). Then, either primary monoclonal mouse anti-HuC/D antiserum (1:500, A21271, RRID:AB_221448, Thermo Fisher Scientific) or primary polyclonal rabbit anti-GFAP antiserum (1:500, Z0334, RRID:AB_10013382, Dako, Glostrup, Denmark) including 0.5% Triton-X and 2% normal goat serum was applied to alternate sections, and the slides were incubated in a humidified chamber for 24 h at 4°C. The sections were visualised with goat anti-mouse IgG secondary antibody, Alexa Fluor 488 conjugated (1.500, A11001, RRID:AB_2534069, Thermo Fisher Scientific) or goat anti-rabbit IgG secondary antibody, Alexa Fluor 488 conjugated (1:500, A11008, RRID:AB_143165, Thermo Fisher Scientific).

#### Image analysis

Section images were captured using a Nikon fluorescent microscope (Eclipse 90i, Nikon, Tokyo, Japan), equipped with a Nikon DXM 1200C camera and NIS-Element 3.0 software.

### Absolute quantification of *rnf38 mRNA* in the brain using real-time PCR

#### Cloning of the *rnf38* gene

Primers for real-time gene expression were designed to amplify a 101 bp fragment of tilapia *rnf38* (GenBank accession number XM 005467588.3) from whole brain cDNA. The primer sequences were as follows: forward primer, 5′-TCTGTGGTCTTCAGTGGTCAA-3′, and reverse primer, 5′- GGGAATGGGTAGGGCATT-3′. The resulting fragment was cloned into a pGEM-T Easy Vector (Promega) by means of RT-PCR cloning method. The primer set was designed to avoid genomic DNA amplification and achieved 97.7% amplification efficiency.

#### Brain tissue preparation for quantitative real-time PCR

Sexually mature males (*n* = 8) were anaesthetised by immersion into 0.02% benzocaine solution (Sigma, St. Louis, MO, USA) prior to decapitation. The brains were rapidly harvested and fresh frozen in frozen section compound on a block of dry ice and stored at −80°C.

#### Brain micro-dissection for quantitative real-time PCR

The brain micro-dissection was performed as previously described with modifications (Maruska et al., 2013). Fresh frozen whole brains were sectioned in coronal plane (60 μm) using a cryostat (Leica CM1860) and briefly thaw-mounted onto uncoated glass microscope slides (Sail Boat Lab Co., Zhejiang, China) and stored at −80°C until microdissection. Brain tissues were collected with a modified 200-μL pipette tip (ExtraGene, Taichung, Taiwan) and 25G needle (Terumo, Tokyo, Japan), and transferred into 200 μL TRIzol reagent (Thermo Fisher Scientific). To prevent cross-contamination and RNA degradation, the needle was cleaned with RNAse-Away (Thermo Scientific), ethanol, and diethyl pyrocarbonate (DEPC)-treated water in successive order between each sample. Tilapia brain atlases (Parhar, 1990; Soga et al., 2005; Ogawa et al., 2016) were used to identify the following brain regions: the telencephalon, preoptic area (including minimal portions of ventral telencephalon), optic tectum, midbrain (including minimal portions of the medullary nuclei of the hindbrain), hypothalamus (including portions of the glomerular, preglomerular, subglomerular, and posterior thalamic nuclei), cerebellum, and hindbrain (**Figure 7A**).

#### RNA extraction and cDNA synthesis

Total RNA was extracted from homogenised microdissected brain tissue with TRIzol reagent according to the manufacturer's instructions. Briefly, 40 μL chloroform was added to the homogenised samples and centrifuged at 12,000 g for 15 min at 4°C to allow separation of the biphasic mixture into a lower red, phenol-chloroform phase and upper colourless, aqueous phase. RNA was precipitated from the aqueous phase by mixing with 100 μL isopropyl alcohol. The samples were then mixed vigorously and centrifuged at 12,000 g for 15 min at 4°C. Upon centrifugation, the supernatant was removed, and the pellet was washed twice with 75% ethanol. After removal of the 75% ethanol and 15 min of drying, the RNA was resolved in 20 μL DEPC-treated water. The total RNA sample (500 ng) was reverse transcribed to cDNA in a reaction mixture of 20 μL containing 1X RT Random Primer, 1X RT buffer, 30U RNase Inhibitor, 75U Multiscribe Reverse Transcriptase with High Capacity cDNA Reverse Transcription Kit (Applied Biosystems) and oligo d(T) primer. The reaction was initiated at 25°C for 10 min for primer annealing, and 37°C for 120 min for reverse transcription, followed by 85°C for 5 min for enzyme inactivation. The cDNA samples were stored at −20°C until use.

#### Absolute quantitative real-time PCR

The amounts of *rnf38* mRNA were determined using quantitative real-time PCR. The PCR reaction was performed in 10-μL duplicate reactions using a SensiFAST SYBR Hi-ROX Kit (Bioline, Taunton, MA, USA) with 0.2 μM each of forward and reverse primers and 1 μL cDNA. The plasmid containing *rnf38* cDNA was serially diluted to concentrations of 10^8^, 10^7^, 10^6^, 10^5^, 10^4^, 10^3^, and 10^2^ copy/μL as standard cDNAs for quantification. Real-time PCR was carried out using 7500 Fast Real-Time PCR system (Applied Biosystems) with conditions of 95°C for 10 min, followed by 40 cycles of 95°C for 15 s and 60°C for 1 min followed by a dissociation step. Single PCR products were verified by melting curve analysis, and the sizes of PCR product were confirmed with agarose gel electrophoresis.

#### Statistical analysis

Data are expressed as the mean values with standard error (SEM) per group.

## Results

### Partial sequence of *rnf38* cDNA

**Figure 1:**
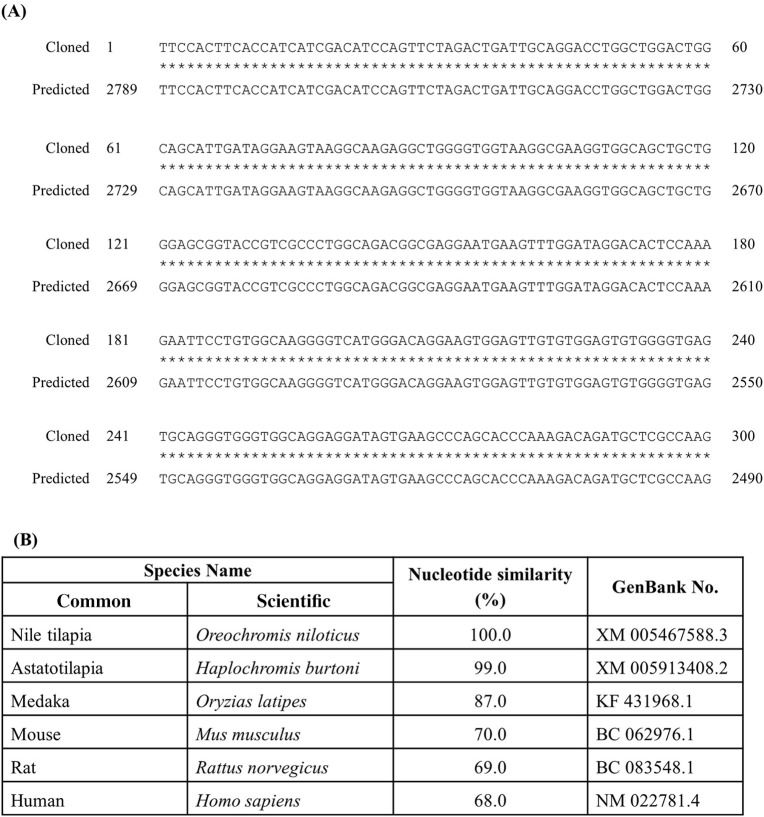
Cloned sequence of tilapia *rnf38* mRNA. **(A)** Alignment of cloned *rnf38* partial sequence with predicted sequence from GenBank (XM 005467588.3; position 2,490–2,789). **(B)** Nucleotide similarity between identified tilapia *rnf38* partial sequences with astatotilapia (*Haplochromis burtoni*), medaka (*Oryzias latipes*), mouse (*Mus musculus*), rat (*Rattus norvegicus*), and human (*Homo sapiens*).

The identified partial length of *rnf38* cDNA consisted of 300 nucleotides spanning 2 exons when compared to the tilapia genome (Figure 1A). The identified partial sequence of *rnf38* cDNA cloned from Nile tilapia showed 100% homology to the predicted sequence in the GenBank (XM 005467588.3) (Figure 1B), and there was a high degree of homology between the partial sequence with the *rnf38* genes of other species such as astatotilapia (*Haplochromis burtoni*), medaka (*Oryzias latipes*), mouse (*Mus musculus*), rat (*Rattus norvegicus*), and human (*Homo sapiens*) (Figure 1B).

### ISH and ICC controls

**Figure 2:**
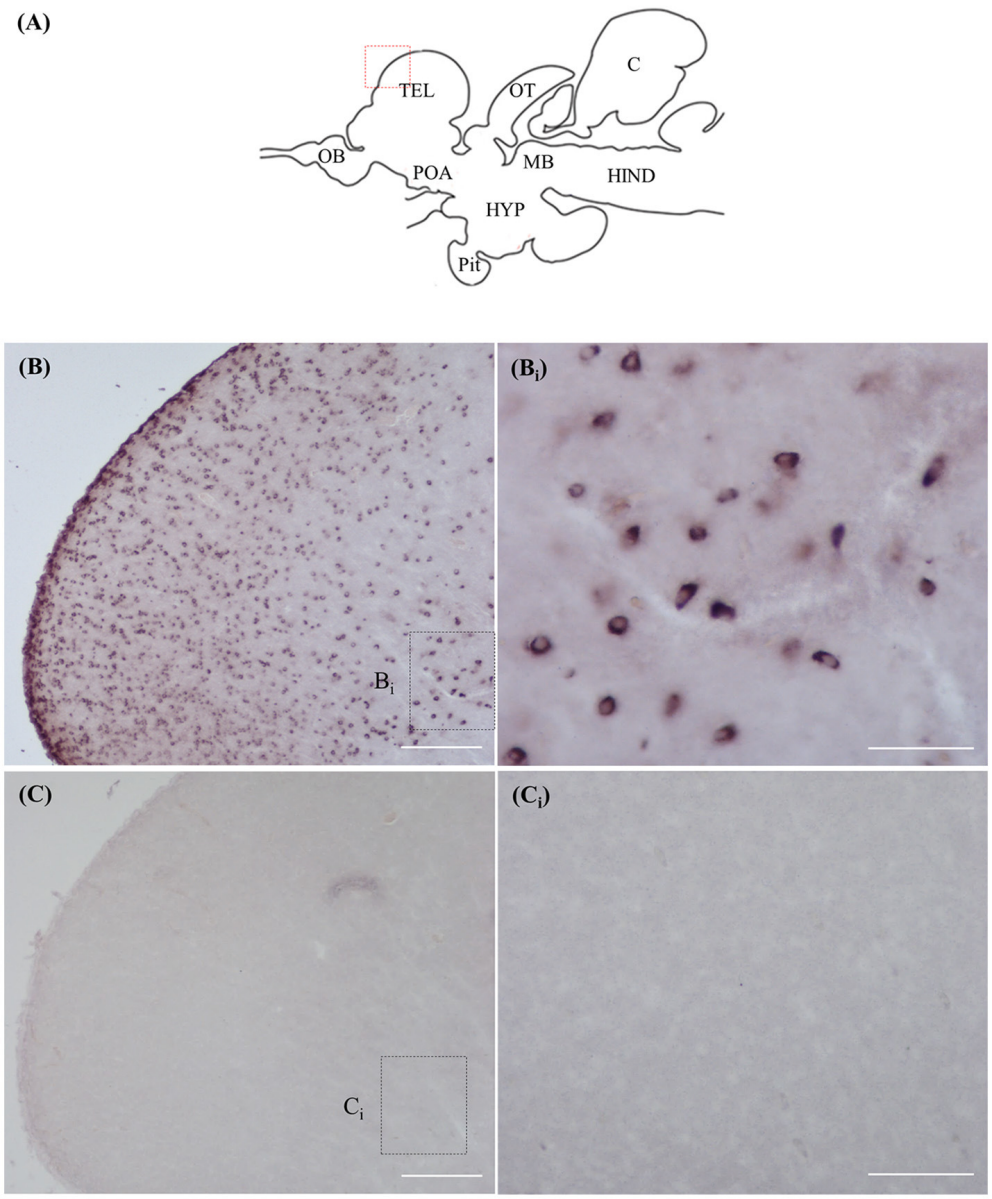
*In situ* hybridisation of *rnf38* in tilapia brain. **(A)** Schematic sagittal diagram of tilapia brain. **(B,B_i_)** *In situ* hybridisation staining with *rnf38* anti-sense RNA probe shows distinct cell-labelling. **(B**_i_**)** shows a higher magnification of the boxed area in **(B)**. **(C,C**_i_**)**. *In situ* hybridisation staining with *rnf38* sense RNA probe shows no hybridisation signal. **C**_i_ shows a higher magnification of the boxed area in **(C)**. Scale bars: **(B,C)**, 200 μm; **(B_i_,C_i_)**, 50 μm.

**Figure 3:**
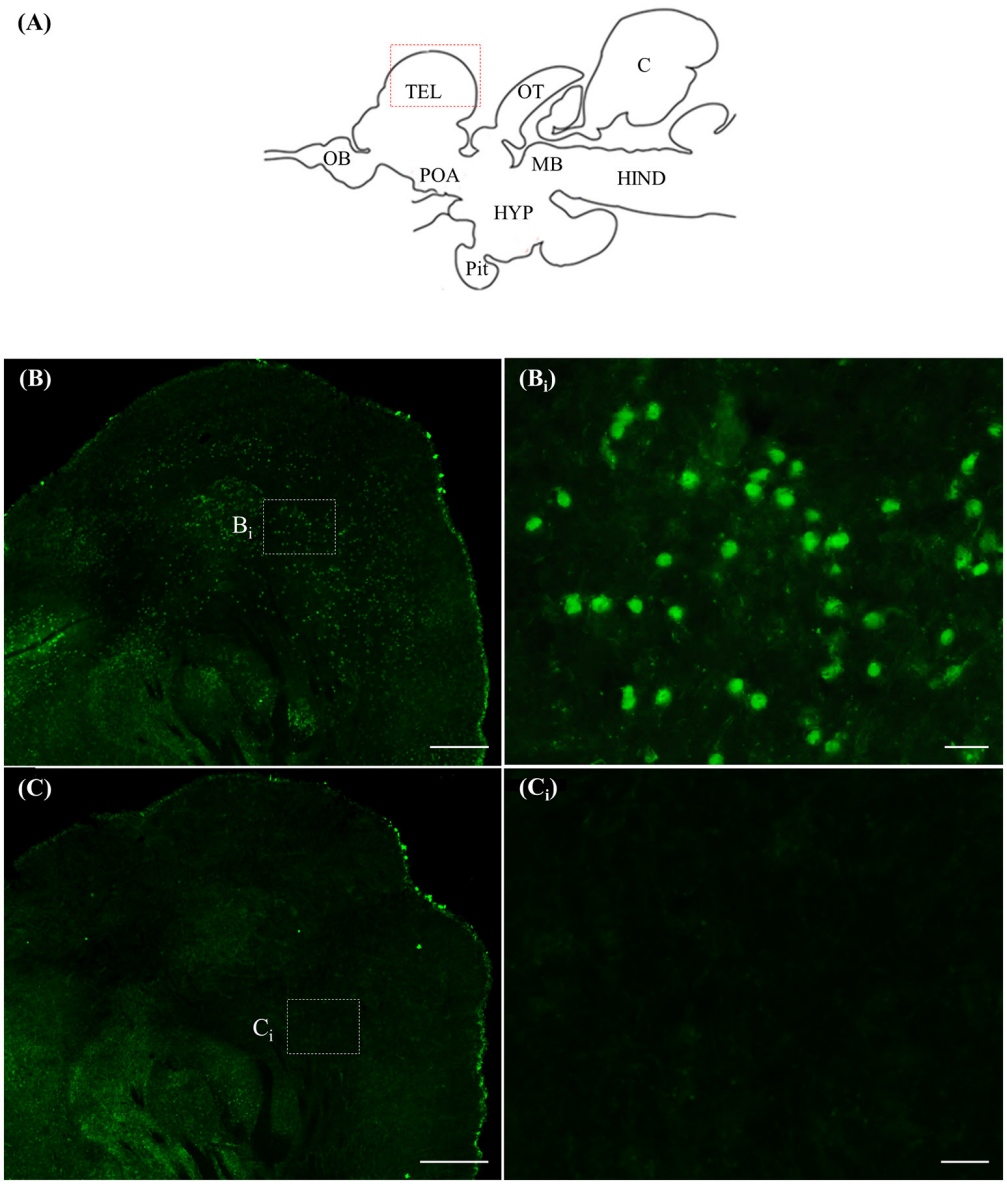
Immunocytochemistry of RNF38 in tilapia brain. **(A)** Schematic sagittal diagram of tilapia brain. **(B,B_i_)** Immunocytochemistry staining with RNF38 antibody shows distinct nuclear-labelling. **(B**_i_**)** shows a higher magnification of the boxed area in **(B). (C,C_i_)** Nuclear staining is completely blocked when the antibody is pre-absorbed with the RNF38 antigen prior to immunocytochemical procedure. **(C**_i_**)** shows a higher magnification of the boxed area in **(C)**. Scale bars: **(B,C)**, 20 μm; **(B_i_,C_i_)**, 20 μm.

Staining with the *rnf38* anti-sense RNA probe showed distinct cell labelling (Figures 2A,B,B_i_), whereas staining with the *rnf38* sense RNA probe showed no hybridisation signal (Figures 2C,C_i_). As for ICC, staining with RNF38 antibody revealed clear nuclear labelling (Figures 3A,B,B_i_), whereas there was no staining with antibody pre-absorbed with the RNF38 antigen (Figures 3C,C_i_).

### Localisation of *rnf38* mRNA and RNF38 protein

**Figure 4:**
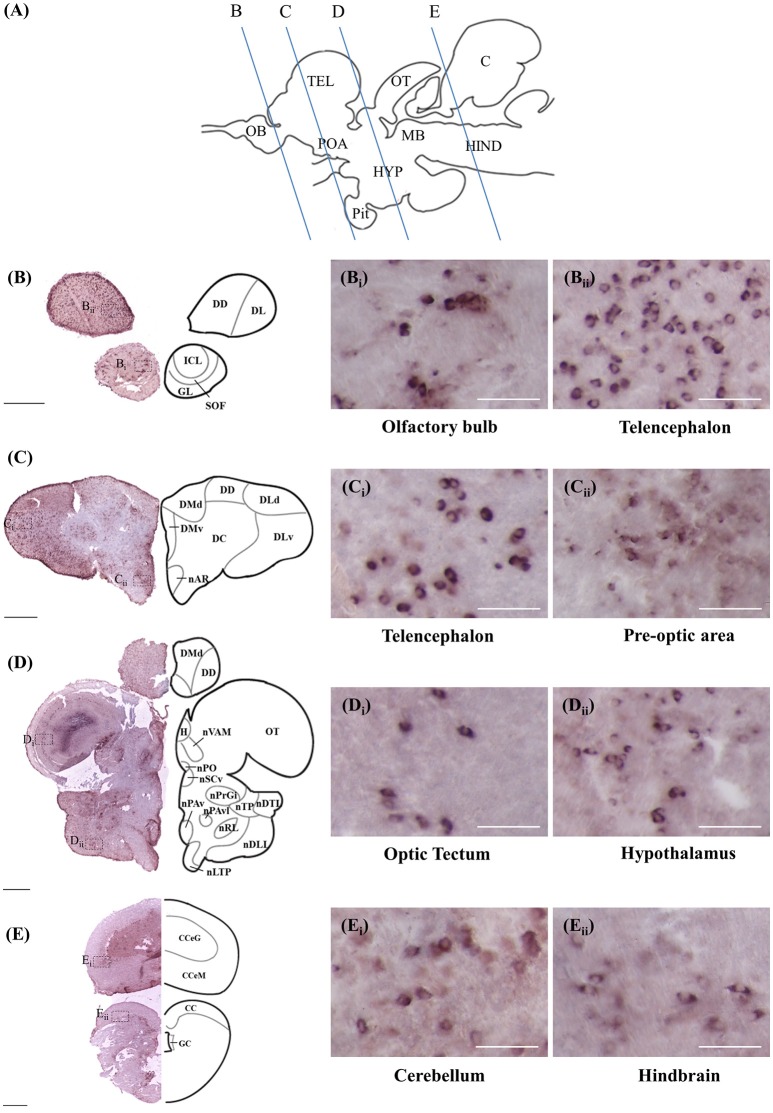
*Rnf38* mRNA expression in the brain of tilapia. **(A)** Schematic sagittal diagram of tilapia brain with approximate locations of coronal sections **(B–E). (B–E)** Representative low magnification photomicrographs of coronal sections are shown on the left and outlined as a mirror image on the right. **(B**_i_,**B**_ii_,**C**_i_,**C**_ii_,**D**_i_,**D**_ii_,**E_i_,E_ii_)** show a higher magnification of the boxed area in **(B–E)** respectively. Scale bars: **(B–E)**, 500 μm; **(B**_i_,**B**_ii_,**C**_i_,**C**_ii_,**D**_i_,**D**_ii_,**E**_i_,**E**_ii_**)**, 50 μm. For abbreviations, see Table 1.

ISH and ICC showed that *rnf38* mRNA and RNF38 protein were expressed throughout the tilapia brain. Figures 4A–E_ii_, 5A–E_ii_ provide an overview of the distributions of *rnf38* hybridisation signals and RNF38 immunoreactivity in the brain (see Table 1).

**Figure 5:**
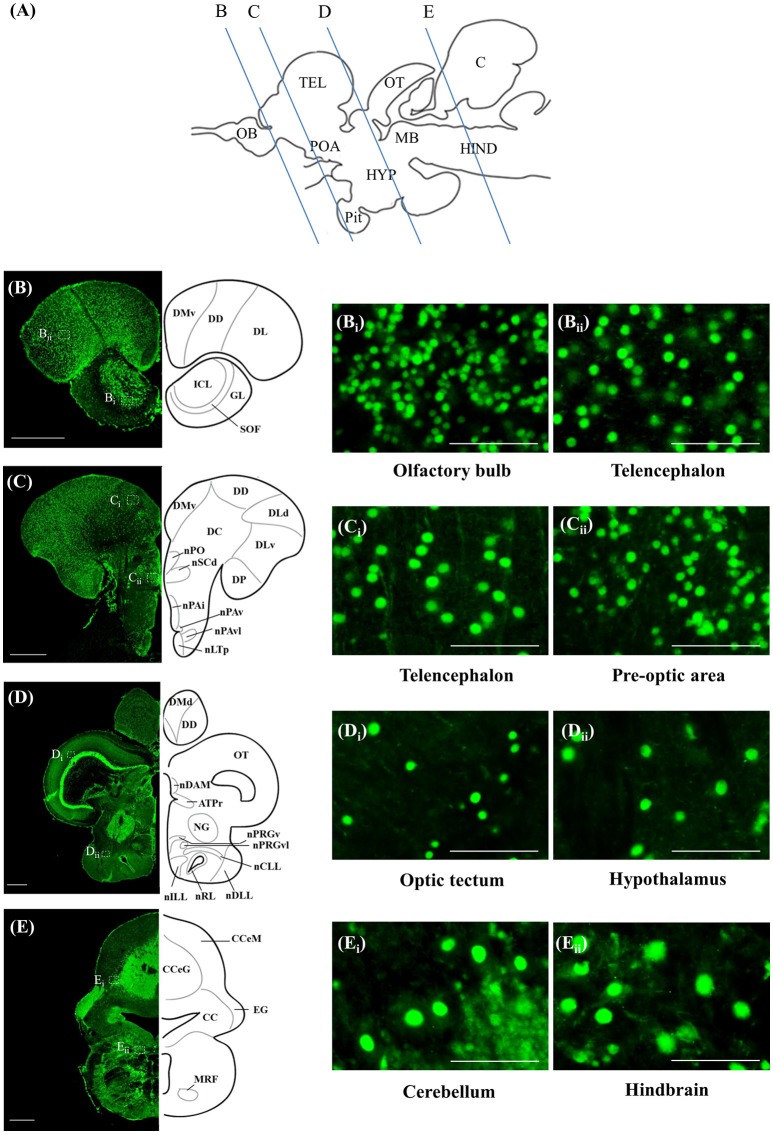
RNF38 protein expression in the brain of tilapia. **(A)** Schematic sagittal diagram of tilapia brain with approximate locations of coronal sections **(B–E)**. **(B–E)** Representative low magnification photomicrographs of coronal sections are shown on the left and outlined as a mirror image on the right. **(B**_i_,**B_ii_,C_i_,C_ii_,D_i_,D_ii_,E_i_,E_ii_)** show a higher magnification of the boxed area in **(B–E)** respectively. Scale bars: **(B–E)**, 500 μm; **(B_i_,B_ii_,C_i_,C_ii_,D_i_,D_ii_,E_i_,E_ii_)**, 50 μm. For abbreviations, see Table 1.

**Table 1:** Distributions of *rnf38* mRNA and protein in the brain of Nile tilapia, *Oreochromis niloticus*.

**Brain region**	**Abbr**.	**Densities of staining**	
		**mRNA**	**Protein**
**TELENCEPHALON**			
**Olfactory bulbs**			
Internal cellular layer	ICL	+ + +	+ + +
Glomerular layer	GL	+ +	+ +
Secondary olfactory fibre layer	SOF	+	+
**Dorsal telencephalon**			
Dorsal zone	DD	+ + +	+ + +
Lateral zone	DL	+ + +	+ + +
Latero-dorsal area	DLd	+ + +	+ + +
Latero-ventral area	DLv	+ + +	+ + +
Central zone	DC	+ + +	+ + +
Medial zone	DM	+ + +	+ + +
Medio-anterior area	DMa	+ + +	+ + +
Medio-dorsal area	DMd	+ + +	+ + +
Medio-ventral area	DMv	+ + +	+ + +
**Ventral telencephalon**			
Dorsal nucleus	Vd	+ + +	+ + +
Ventral nucleus	Vv	+ + +	+ + +
Supracommissural nucleus	Vs	+ + +	+ + +
Entopeduncular nucleus	nE	+ + +	+ + +
**DIENCEPHALON**			
**Preoptic area**			
Anterior preoptic recess nucleus	nAR	+ + +	+ + +
Nucleus preopticus	nPO	+ + +	+ + +
Suprachiasmatic nucleus	nSC	+ + +	+ + +
Dorsal zone	nSCd	+ + +	+ + +
Ventral zone	nSCv	+ + +	+ + +
**Epithalamus**			
Habenula	H	+ + +	+ + +
Epiphysis	EP	+	+
**Dorsal thalamus**			
Posterior zone	DP	+ + +	+ + +
Medial zone	nDAM	+ + +	+ + +
Lateral zone	nDVL	+ +	+ +
Glomerular nucleus	NG	+ +	+ +
**Ventral thalamus**			
Lateral zone	nVAL	+ +	+ +
Medial zone	nVAM	+ + +	+ + +
**Posterior tuberculum**			
Posterior tuberal nucleus	nPT	+ + +	+ + +
Posterior tuberal area			
Rostral zone	ATPr	+ + +	+ + +
Lateral zone	ATPl	+ +	+ +
Preglomerular nucleus			
Anterior zone	nPrGa	+ +	+ +
Dorso-lateral subdivision	nPrGdl	−	−
Inferior zone	nPrGi	+ + +	+ + +
Medial subdivision	nPrGm	+ + +	+ + +
Superior subdivision	nPrGs	+ + +	+ + +
Ventral subdivision	nPrGv	+ + +	+ + +
Ventro-lateral subdivision	nPrGvl	+ +	+ +
Posterior thalamic nucleus	PT	+ + +	+ + +
Subglomerular nucleus	nSbG	+ +	+ +
Posterior thalamic nucleus	nTP	+ + +	+ + +
**Periventricular area**			
Anterior periventricular nucleus			
Dorsal zone	nPAd	+ + +	+ + +
Dorso-lateral subdivision	nPAdl	+ +	+ +
Intermedial zone	nPAi	+ + +	+ + +
Ventral zone	nPAv	+ +	+ +
Ventro-lateral subdivision	nPAvl	+ + +	+ + +
Posterior periventricular nucleus			
Dorsal zone	nPPd	+ + +	+ + +
Medial zone	nPPm	+ + +	+ + +
Ventral zone	nPPv	+ + +	+ + +
**Hypothalamus**			
Anterior tuberal nucleus	nAT	+ +	+ +
Lateral tuberal nucleus			
Anterior zone	nLTa	+ + +	+ + +
Lateral zone	nLTl	+ + +	+ + +
Posterior zone	nLTp	+ + +	+ + +
Rostral zone	nLTr	+ + +	+ + +
Ventral zone	nLTv	+ +	+ +
Periventricular hypothalamus			
Ventral zone	Hv	+ + +	+ + +
Dorsal zone	Hd	+ + +	+ + +
Paraventricular organ	PVo	+ + +	+ + +
Diffuse nucleus of the lateral lobe	nDLL	+ + +	+ + +
Diffuse nucleus of the lateral tori	nDTL	+ + +	+ + +
Intermediate nucleus of the lateral lobe	nILL	+ + +	+ + +
Central nucleus of the lateral lobe	nCLL	+ + +	+ + +
Lateral recess nucleus	nRL	+ + +	+ + +
Posterior recess nucleus	nRP	+ + +	+ + +
**Synencephalon**			
Nucleus of the medial longitudinal fascicle	nMLF	+ + +	+ + +
**Pretectum**			
Pretectal area	AP	+ + +	+ + +
Pretectal nucleus			
Dorsal zone	nPCd	+ + +	+ + +
Ventral zone	nPCv	+ +	+ +
Fasciculus retroflexus	FR	+	+
**MESENCEPHALON**			
*Optic tectum*	OT	+ + +	+ + +
**Semicircular torus**			
Semicircular torus (layer 2)	TS2	+ + +	+ + +
Semicircular torus (layer 3)	TS3	+ +	+ +
**Tegmentum**			
Dorsal tegmental nucleus	DTN	+ +	+ +
Rostral tegmental nucleus	RT	+ +	+ +
Perilemniscal nucleus	pL	+ + +	+ + +
Oculomotor nucleus	NIII	+ + +	+ + +
Medial nucleus	MR	+ + +	+ + +
Superior nucleus	SR	+ + +	+ + +
**RHOMBENCEPHALON**			
**Cerebellum**			
Valvula cerebelli			
Lateral division	Val	+	+
Medial division	Vam	+	+
Granular zone of the corpus cerebellum	CCeG	−	−
Molecular zone of the corpus cerebellum	CCeM	+ +	+ +
Eminentia granularis	EG	+ +	+ +
**Medullary nuclei**			
Crista cerebellaris	CC	−	+
Superior reticular formation	SRF	+ + +	+ + +
Lateral reticular formation	LRF	+ +	+ +
Medial or intermediate reticular formation	MRF	+ + +	+ + +
Central gray	GC	+ +	+ +
Lateral valvular nucleus	nLV	+ + +	+ + +
Lateral longitudinal fascicle	LLF	−	−
Vascular lacuna of area postrema	VAS	−	−

*+ + +, high; + +, moderate; +, low; −, absent*.

#### Telencephalon

In the olfactory bulb, there was very dense staining of positively labelled cells in the internal cell layer (ICL) (Figures 4B, 5B). Staining of positively labelled cells was also observed in the glomerular layer (GL) and secondary olfactory fibre layer (SOF) of the olfactory bulb (Figures 4B, 5B). In the dorsal telencephalon, there was robust staining of positively labelled cells in almost all subdivisions including the dorsal, lateral, and medial zones (DD, DL, and DM, respectively) (Figures 4B–D, 5B–D). However, staining intensity in the medio-ventral area of the dorsal telencephalon (DMv) was weaker compared to other telencephalic areas (Figures 4C, 5B–C). Although strong staining of positively labelled cells was observed in the central area of the dorsal telencephalon (DC), the distribution was quite diffuse (Figures 4C, 5C). Heavy staining was observed in all subdivisions of the ventral telencephalon including the dorsal, ventral, supracommissural, and entopeduncular nuclei (Vd, Vv, Vs, and nE, respectively) (Table 1).

#### Diencephalon

The preoptic area (POA) had very intense staining, specifically in the anterior preoptic recess nucleus (nAR), nucleus preopticus (nPO), and dorsal and ventral zones of the suprachiasmatic nucleus (nSCd and nSCv) (Table 1, Figures 4C–D, 5C). Caudal to the POA, several subdivisions of the dorsal thalamus showed positively staining including the posterior, medial, and lateral zones (DP, nDAM, and nDAL, respectively) and glomerular nucleus (NG) (Table 1, Figure 5D). Strong staining was present in the lateral and medial zones of the ventral thalamus (nVAL and nVAM) (Table 1, Figure 4D). In the epithalamus, intense signals were present in the habenula (H) (Table 1, Figure 4D). Other subdivisions of the epithalamus, such as the epiphysis (EP), also contained positively labelled cells (Table 1).

Positively labelled cells were identified in almost all subdivisions of the posterior tuberculum, which includes the posterior tuberal nucleus (nPT), rostral and lateral zones of posterior tuberal area (ATPr and ATPl), and anterior, inferior, medial, superior, ventral, and ventro-lateral subdivisions of the preglomerular nucleus (nPrGa, nPrGi, nPrGm, nPrGs, nPrGv, and nPrGvl, respectively) (Table 1, Figures 4D, 5D). Strong staining was also observed in other subdivisions of posterior tuberculum, which includes the posterior thalamic nucleus (PT), subglomerular nucleus (nSbG), and posterior thalamic nucleus (nTP) (Table 1). Hybridisation signals and immunoreactivity were absent in the dorso-lateral subdivision of preglomerular nucleus (nPrGdl) (Table 1).

Strong staining of positively labelled cells was found throughout the hypothalamus. Positively labelled cells were present in the anterior tuberal nucleus (NAT), and anterior, lateral, posterior, and rostral zones of the lateral tuberal nucleus (nLTa, nLTl, nLTp, and nLTr, respectively) of the hypothalamus (Table 1, Figure 4D). Within the periventricular hypothalamus, intense staining was observed in the ventral and dorsal zones (Hv and Hd) (Table 1). Strong staining was also found in diffuse nuclei of the lateral lobe and lateral tori (nDLL and nDTL), intermediate and central nucleus of lateral lobe (nILL and nCLL), and lateral and posterior recess nuclei (nRL and nRP) (Table 1, Figures 4D, 5D). Positively labelled cells were also present in the anterior periventricular nuclei including the dorsal, dorso-lateral, intermedial, ventral, and ventro-lateral parts (nPAd, nPAdl, nPAi, nPAv, and PAvl, respectively) (Table 1, Figure 4D). Staining was also observed in the posterior periventricular nuclei including the dorsal, medial, and ventral parts (nPPd, nPPm, and nPPv) (Table 1).

Strong staining was present in the nucleus of the medial longitudinal fascicle (nMLF), pretectal area (AP), and pretectal nucleus including the dorsal and ventral regions (nPCd and nPCv, respectively) (Table 1). Signals were also present along the border of the fasciculus retroflexus fibre tract (FR) (Table 1).

#### Mesencephalon

Intense staining of positively labelled cells was observed in the optic tectum (OT) (Figures 4D, 5D). Strong signals were observed in the layers 2 and 3 of the semicircular torus (TS2 and TS3), dorsal and rostral tegmental nucleus (DTN and RT), and perilemniscal nucleus (pL) (Table 1). Positive cells were also present in the oculomotor nucleus (NIII), which includes the medial and superior nuclei of the nervus oculomotorius (MR and SR) (Table 1).

#### Rhombencephalon

There were relatively fewer positively labelled cells in the rhombencephalon compared to telencephalon, diencephalon, and mesencephalon. Within the cerebellum, staining was observed in the ganglionic layer between the molecular and granular layers of the corpus cerebellum (CCeM and CCeG, respectively) (Figures 4E, 5E). Staining was also observed in other subdivisions of the cerebellum: the lateral and medal division of the valvula cerebelli and the eminentia granularis (Val, Vam, and EG, respectively) (Table 1). However, in the crista cerebellaris (CC) of the medullary nuclei, despite minimal RNF38 immunoreactive labelling, no hybridisation signals were observed (Figures 4E, 5E). Caudally in the rhombencephalon, other medullary nuclei also had patches of strong staining of positively labelled cells, specifically in the superior reticular formation, lateral reticular formation, and medial or intermediate reticular formation (SRF, LRF, and MRF, respectively) (Table 1). Strong staining was also observed in the central gray (CG) and lateral valvular nucleus (nLV) (Table 1, Figure 4E). No signal was observed in the lateral longitudinal fascicle (LLF) or vascular lacuna of the area postrema (VAS) (Table 1).

### Type of RNF38-expressing cells

**Figure 6:**
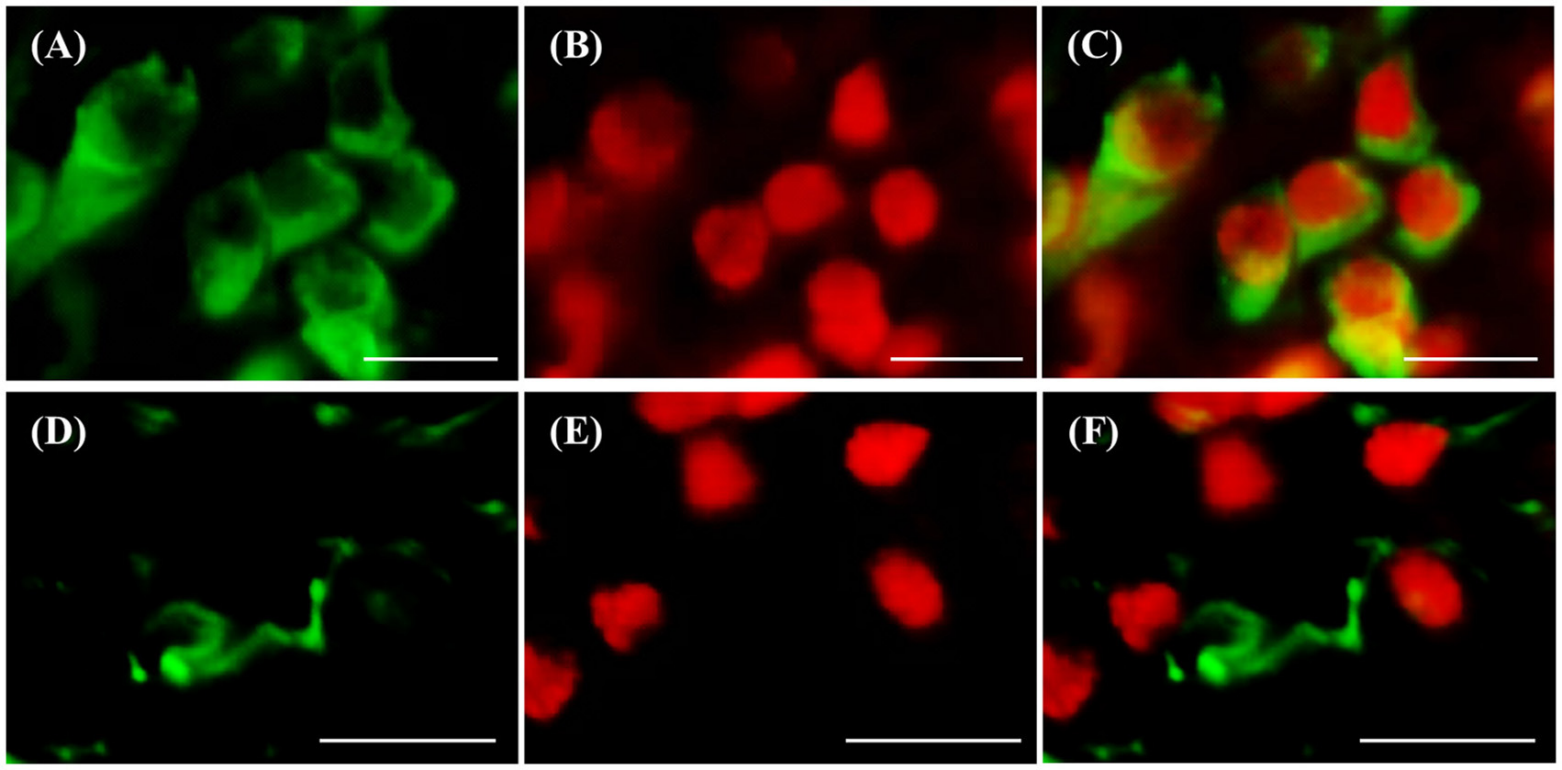
Double-labelling immunocytochemistry of RNF38 protein with neuronal marker HuC/D or glial marker GFAP. **(A–C)** Neuronal marker HuC/D (**A**, green), RNF38 peptide (**B**, red), merged image **(C)**. **(D–F)** Glial marker GFAP (**D**, green), RNF38 protein (**E**, red) and merged image (**F**). Scale bars: **(A–F)**, 10 μm.

d-ICC for RNF38 and the neuronal marker HuC/D showed that most RNF38 protein, although not all, was expressed in neuronal nuclei (Figures 6A–C). Correspondingly, d-ICC for RNF38 and GFAP showed that RNF38 was absent in astroglial cells (Figures 6D–F).

### Levels of *rnf38* mRNA in the brain

**Figure 7:**
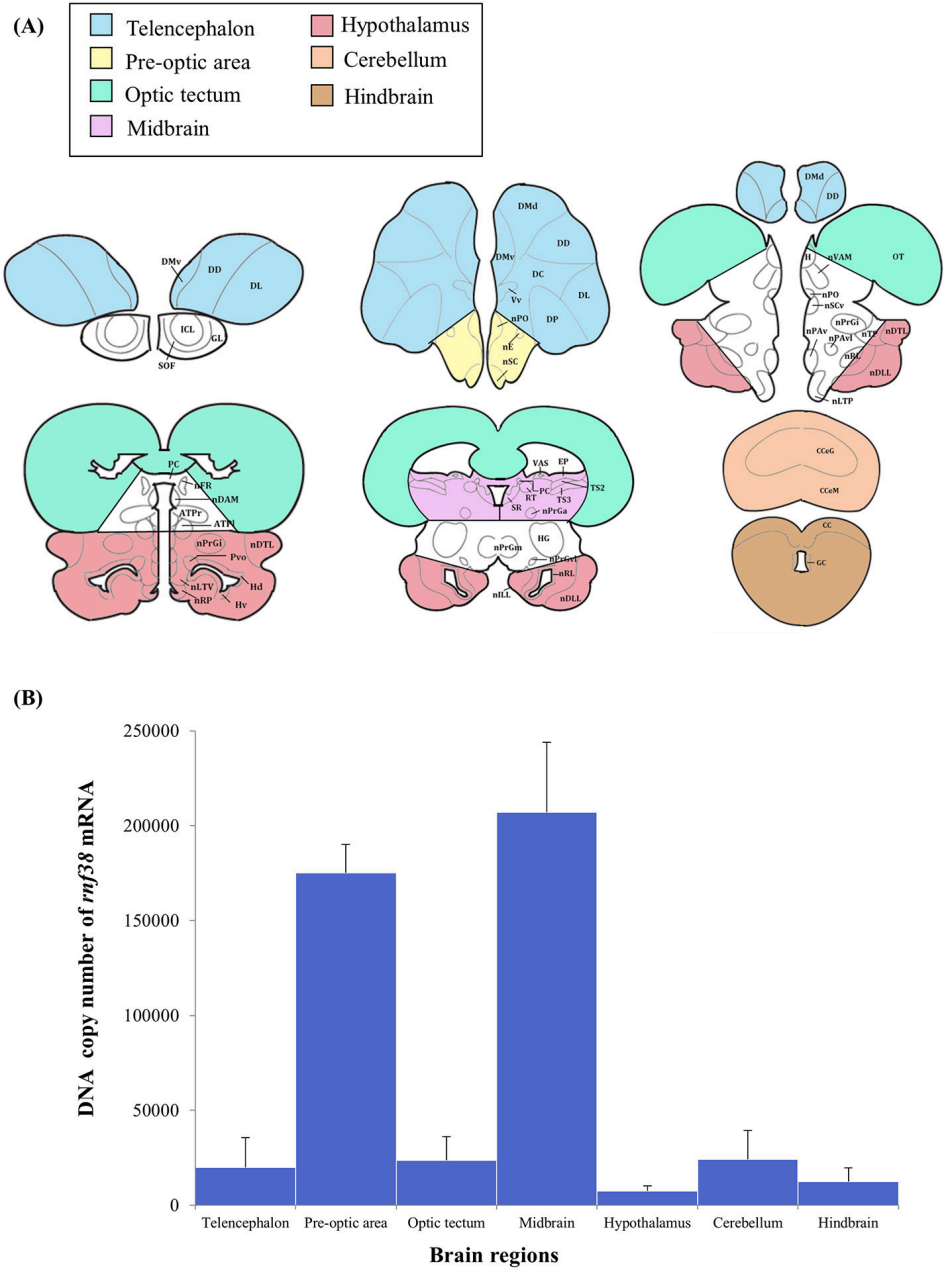
Distribution of *rnf38* mRNA in the telencephalon, preoptic area, optic tectum, midbrain, hypothalamus, cerebellum and hindbrain of tilapia. **(A)** Brain areas collected during microdissection (telencephalon, *n* = 5; preoptic area, *n* = 6; optic tectum, *n* = 8; midbrain, *n* = 7; hypothalamus, *n* = 6; cerebellum, *n* = 5; and hindbrain, *n* = 5) for *rnf38* gene expression study. **(B)** DNA copy number of *rnf38* mRNA in telencephalon, optic tectum, hypothalamus, cerebellum and hindbrain of male tilapia. Data represent the means ± SEM.

Real-time PCR was performed to examine *rnf38* gene expression in seven brain regions: the telencephalon, preoptic area, optic tectum, midbrain, hypothalamus, cerebellum and hindbrain. The highest levels of *rnf38* mRNA were detected in the midbrain, followed by the preoptic area, cerebellum, optic tectum, telencephalon, hindbrain and hypothalamus (Figure 7B). The regional distribution of *rnf38* mRNA detected using real-time PCR corresponded well with that of *rnf38* mRNA visualised by ISH.

## Discussion

### *rnf38* gene sequence

To the best of our knowledge, we used genomic cloning to successfully obtain a partial sequence of *rnf38* cDNA in the Nile tilapia. It showed 100% homology to the predicted sequences in GenBank (XM 005467588.3), thus confirming the partial sequence of tilapia *rnf38*. Comparison with *rnf38* genes of other species such as *Haplochromis burtoni* (99%, GenBank XM 005913408.2), *Oryzias latipes* (87%, GenBank KF 431968.1), *Mus musculus* (70%, GenBank BC 062976.1), *Rattus norvegicus* (69%, GenBank BC 083548.1), and *Homo sapiens* (68%, GenBank NM 022781.4) showed high homology. This indicates that the gene is significantly conserved from an evolutionary perspective, suggesting functional relevance.

### Expression of *rnf38* mRNA and RNF38 protein in the brain

The present study examined the distribution of positively labelled cells in the brain of the Nile tilapia using ISH and ICC. Positively labelled cells in ISH are indicative of *rnf38* expression, while those from ICC are indicative of the presence of the protein. Several lines of evidence show that the RNA probe and antibody can specifically identify the expression of *rnf38* mRNA and its peptide, respectively. Firstly, the labelled cells were not observed in sections labelled with the sense probe, and pre-absorption of the antibody with antigen blocked all immunostaining. Second, there was good correspondence between *rnf38* mRNA expression patterns and RNF38 protein immunoreactivity. As specificity has been demonstrated for the RNF38 probe and antibody, they will be useful for future investigations of the roles of RNF38 in social behaviour. Such studies may include examining changes in *rnf38* mRNA levels during social defeat in Nile tilapia. The antibody will also be useful for double-labelling experiments with serotonergic neurons to clarify the neurochemical properties of RNF38 cells.

ISH and ICC showed that *rnf38* mRNA and protein are widely distributed throughout the brain. In the quantitative assay, real-time PCR demonstrated that the *rnf38* mRNA expression corresponded to the distribution of *rnf38* mRNA as visualised by ISH. Via real-time PCR, the expression of *rnf38* mRNA was previously reported in the telencephalon, optic tectum, hypothalamus, cerebellum, and hindbrain of the medaka fish (Moriya et al., 2015), as well as in the hypothalamus of male mice (Fu et al., 2006). Widespread RNF38 expression throughout the brain indicates its potential involvement in a variety of cellular functions.

ISH revealed substantially more positively labelled cells in the telencephalon compared to the hindbrain; however, real-time PCR data showed little difference between the two regions. This discrepancy may be due to that the real-time PCR data reflects *rnf38* mRNA copy number per 500 ng of RNA, whereas ISH indicates the cells expressing *rnf38* mRNA rather than the amount of mRNA. It is highly possible that to compensate for the smaller number of cells compared to the telencephalon, each *rnf38*-positive cell in the hindbrain expresses more mRNA per cell. Notably, the discrepancy is not due to absence of normalisation: expression of the housekeeping gene *B-actin* was stable in all brain regions (data not shown).

In the present study, real-time PCR showed high *rnf38* mRNA expression in the midbrain, an area implicated in monoamine synthesis by serotonergic neurons (Loveland et al., 2014). Furthermore, chronic treatment of the medaka fish with the antidepressant citalopram up-regulated brain expression of *rnf38* (Moriya et al., 2015), as did similar treatment of male mice (Soga et al., unpublished observations). This suggests a relationship between RNF38 and the serotonergic system. Therefore, it is possible that *rnf38* could potentially regulate the serotonergic system or vice versa. *Rnf38* mRNA was highly expressed in the preoptic area, which suggests its potential role in behaviour and reproduction (Liu et al., 1997; Foran and Bass, 1999; Goodson and Bass, 2000; Floody et al., 2011; Floody, 2014).

### Functional considerations

The widespread expression of RNF38 throughout the brain indicates its potential involvement in a variety of cellular functions. Previous studies have shown that RNF38 is an E3 ubiquitin ligase, and protein ubiquitination is a highly regulated cellular process (Deshaies and Joazeiro, 2009; Sheren and Kassenbrock, 2013). Furthermore, RNF38 can interact with and activate the ubiquitin-conjugating enzyme E2 (Buetow et al., 2015), thus potentially acting as an intrinsic factor that mediates ubiquitination via the RING finger domain. Since RNF38 is a nuclear protein capable of ubiquitinating p53 (Sheren and Kassenbrock, 2013), it is therefore a potential regulator of p53 signalling. In addition, our results show that RNF38 protein is localised in the nucleus. The presence of a coiled-coil domain near the RING finger domain of *rnf38*, a region involved in protein-protein/DNA interaction, predicts that RNF38 possesses DNA-binding and transcriptional activities (Morett and Bork, 1999; Moriya et al., 2015). Our results also provide the first evidence that RNF38 is expressed in neurons, suggesting that it possesses neuronal activity.

## Conclusion

In summary, we successfully identified the partial sequence of *rnf38*, and localised *rnf38* mRNA and RNF38 protein in the brain of Nile tilapia using ISH and ICC respectively. The *rnf38* mRNA and its peptide were distributed throughout the tilapia brain. RNF38 expression was highest in the preoptic area and midbrain. We also successfully showed that the majority of RNF38 protein, although not all, was expressed in neuronal nuclei. This work established the neuroanatomical distribution of RNF38 in the tilapia fish brain. Based on its widespread, differential localisation, RNF38 appears to have a broad, and possibly diverse influence on central nervous system function.

## Author contributions

KC conducted all experiments and analysed the data together with TS. KC wrote this manuscript. TS and IP designed the experiment, did data analysis and edited the manuscript together. TS and IP received a research grant from Malaysia government for this project.

### Conflict of interest statement

The authors declare that the research was conducted in the absence of any commercial or financial relationships that could be construed as a potential conflict of interest.
